# The Healthy Lifestyles Programme (HeLP), a novel school-based intervention to prevent obesity in school children: study protocol for a randomised controlled trial

**DOI:** 10.1186/1745-6215-14-95

**Published:** 2013-04-04

**Authors:** Katrina M Wyatt, Jennifer J Lloyd, Charles Abraham, Siobhan Creanor, Sarah Dean, Emma Densham, Wendy Daurge, Colin Green, Melvyn Hillsdon, Virginia Pearson, Rod S Taylor, Richard Tomlinson, Stuart Logan

**Affiliations:** 1 Institute of Health Services Research, University of Exeter Medical School (formerly Peninsula College of Medicine and Dentistry), Veysey Building, Salmon Pool Lane, Exeter, Devon EX2 4SG, UK; 2 School of Computing and Mathematics, Faculty of Science and Technology, Room 301, Biostatistics, ITTC Building, Tamar Science Park, Plymouth, Devon PL6 8BX, UK; 3 Sport and Health Sciences, College of Life and Environmental Sciences, University of Exeter St Luke's Campus, Heavitree Road, Exeter, EX1 2LU, UK; 4 Royal Devon and Exeter Hospital, Royal Devon & Exeter NHS Trust, Department of Child Health Barrack Road, Exeter, EX2 5DW, UK; 5 Directorate of Public Health, NHS Devon Commissioning Headquarters, County Hall, Topsham Road, Exeter, EX2 4QL, UK; 6 Isca College, Earl Richards Rd, Exeter, Devon, EX2 6AP, UK; 7 St Leonard's Primary School, St Leonards Rd, Exeter, Devon, EX2 4NQ, UK

**Keywords:** Childhood obesity, School-based, Cluster trial, 9 to 10 year olds, Engagement, Parental involvement, Randomised controlled trial

## Abstract

**Background:**

Over the last three decades there has been a substantial increase in the proportion of children who are overweight or obese. The Healthy Lifestyles Programme (HeLP) is a novel school-based intervention, using highly interactive and creative delivery methods to prevent obesity in children.

**Methods/Design:**

We describe a cluster randomised controlled trial to evaluate the effectiveness and cost effectiveness of HeLP. The intervention has been developed using intervention mapping (involving extensive stakeholder involvement) and has been guided by the Information, Motivation, Behavioural Skills model. HeLP includes creating a receptive environment, drama activities, goal setting and reinforcement activities and runs over three school terms. Piloting showed that 9 to 10 year olds were the most receptive and participative. This study aims to recruit 1,300 children from 32 schools (over half of which will have ≥19% of pupils eligible for free school meals) from the southwest of England. Participating schools will be randomised to intervention or control groups with baseline measures taken prior to randomisation. The primary outcome is change in body mass index standard deviation score (BMI SDS) at 24 months post baseline. Secondary outcomes include, waist circumference and percent body fat SDS and proportion of children classified as overweight or obese at 18 and 24 months and objectively measured physical activity and food intake at 18 months. Between-group comparisons will be made using random effects regression analysis taking into account the hierarchical nature of the study design. An economic evaluation will estimate the incremental cost-effectiveness of HeLP, compared to control, from the perspective of the National Health Service (NHS)/third party payer. An in-depth process evaluation will provide insight into how HeLP works, and whether there is any differential uptake or engagement with the programme.

**Discussion:**

The results of the trial will provide evidence on the effectiveness and cost effectiveness of the Healthy Lifestyles Programme in affecting the weight status of children.

**Trial registration:**

ISRCTN15811706

## Background

In recent years Britain has become a nation where adult overweight is the norm with Foresight modelling predicting that 60% of adult men, 50% of adult women and 25% of all children under 16 could be obese by 2050. The financial impact to society at current prices is estimated to become an additional £45.5 billion per year by 2050 with a seven-fold increase in National Health Service (NHS) costs alone [1]. Obesity in children and adolescents is associated with a range of adverse metabolic and cardiovascular traits [2,3]: exacerbation of asthma [4], poor self-esteem [5] and an increased likelihood of being obese in adulthood [6,7]. Prevention, especially in children, is universally viewed as the best approach; however, evidence for effective interventions is scarce.

The most recent Health Survey for England [8] reports that 16.1% of boys and 15.3% of girls aged 2 to 15 were obese with 15.4% of boys and 12.9% of girls classified as overweight, and the National Child Measurement Programme [9] data report that more than one in five 5 to 6 year olds and one in three 10 to 11 year olds are either overweight or obese.

The relative contribution of physical activity, sedentary activity, and diet to the development of obesity in children is unclear, partly because the variables are difficult to measure and the balance of energy is complex [10,11]. In addition, these lifestyle factors also interact with genetic factors affecting people’s propensity to gain weight, thus creating a highly individualised complex equation of factors leading to the development of obesity. However, prolonged periods of sitting (for example, TV viewing/screen-based activity) [12]; low levels of physical activity [13]; parents’ inactivity [14]; and high consumption of dietary fat, carbohydrates, and sweetened fizzy drinks [15-17] have been identified as common and modifiable risk factors that can be easily targeted in school-based interventions.

It is unsurprising that most childhood prevention programmes to date have been situated within the school particularly when their existing organisational, social and communication structures provide opportunities for regular health education and the possibility of a health-promoting environment. In addition, they have the potential to reach children and their families across the social spectrum, however, despite the increasing number of school-based interventions to prevent obesity in children, results continue to be inconsistent and it is still unclear what the necessary conditions are that lead to the sustained behaviour change necessary to affect weight status.

Brown and Summerbell’s [18] review of controlled trials of school-based interventions identified nine combined diet and physical activity interventions that showed significant improvements in mean BMI in favour of the intervention; however, only five of these studies followed up the children for longer than 12 months, considered by the National Institute for Clinical Effectiveness (NICE) [19] to be the minimal length of follow up to reasonably assess long-term outcomes. The authors of the review concluded that there was insufficient evidence to determine the effectiveness of dietary interventions alone, but suggested that interventions that increase activity and reduce sedentary behaviour may help children to maintain a healthy weight, although results were short term and inconsistent.

Since this review, the results of other large-scale school-based trials have been published. For example The CHILDREN study [20], a one-year intervention for 10 to 11 year old children, based on the Theory of Planned Behaviour [21] and involving parental support, showed a significant difference in BMI in favour of the intervention at one-year follow-up. The HEALTHY Study group [22] developed a three-year school-based intervention for 11 to 14 year olds using social marketing and building skills; however, the results did not show a significant difference between the control and intervention groups in the primary outcome (the combined prevalence of overweight and obesity) at the end of the three-year study. The Dutch Obesity Intervention in Teenagers Trial (DOiT) [23], which used education and environmental change showed significant differences in favour of the intervention for skinfold measures but not for BMI at 20-month follow-up.

A recent review by Khambalia and colleagues [24] examined the quality of evidence and findings from existing systematic reviews and meta-analyses of school-based programmes in the control and prevention of childhood obesity published between 1990 and 2010. All of the reviews recognised that studies were heterogeneous in design, participants, intervention and outcomes. Intervention components in the school setting associated with a significant reduction of weight in children included long-term interventions with combined diet and physical activity and a family component. Khambalia and colleagues concluded that, as no single intervention will fit all school populations, further high quality research needs to focus on identifying specific programme characteristics predictive of success.

Peters *et al*. [25] carried out a review of reviews of effective elements of school health promotion across behavioural domains (substance abuse, sexual behaviour and nutrition). Five effective elements were highlighted across all three domains: use of theory, addressing social influences (especially social norms), addressing cognitive behavioural skills, training of facilitators and multiple components. The authors concluded that these elements should be primary candidates to include in programmes targeting these behaviours. In addition, the Foresight review [26] and recent research suggest that engaging parents and offering them strategies through which they can directly (through parenting) or indirectly (through the creation of supportive environments) foster the development of healthy eating and activity behaviours among their children/family is crucial in initiating and sustaining behavioural change [27,28]. It is also important to use delivery methods that engage the children sufficiently to be motivated to change and, crucially, to engage their parents [29,30]. A systematic review of school-based drug-prevention programmes [31] showed that the most effective programmes used interactive delivery methods, used peer leaders and focussed on affecting peer norms, yet despite its potential to empower and engage children in particular, only a few school-based health promotion programmes have primarily or solely involved interactive drama as a delivery method [29,30]. Initial results from an exploratory trial showed that schools, children and their families found the trial design and the intervention feasible and acceptable. Moreover, at 18 months follow-up, intervention children had fewer ‘negative food markers’, consumed less energy dense snacks and more healthy snacks, had more ‘positive food markers’, had lower mean TV/screen time and spent more time doing moderate to vigorous physical activity each day than children in the control schools. Intervention children had lower anthropometric measures at 18 and 24 months than control children, with larger differences at 24 months than at 18 months for all measures except percentage body fat SDS [32].

### Aim

The aim of this cluster randomised controlled trial (RCT) is to determine the effectiveness and cost-effectiveness of the Healthy Lifestyles Programme (HeLP) in preventing overweight and obesity in children.

#### Specific objectives

1. To assess the effectiveness of the Healthy Lifestyles Programme (HeLP), in children aged 9 to 10 years, by comparing in intervention and control schools:

a. BMI SDS at 18 and 24 months (primary outcome)

b. Waist Circumference SDS at 18 and 24 months

c. Percentage Body Fat SDS at 18 and 24 months

d. Proportion of children classified as underweight, overweight and obese at 18 and 24 months

e. Physical activity at 18 months

f. Food intake at 18 months

2. To assess the costs of HeLP and its cost-effectiveness versus usual practice

3. To conduct a mixed-methods process evaluation and mediational analysis to explore the way the Programme worked (that is, how it was delivered, taken up, and experienced, and what the behavioural mediators of change are).

## Methods/Design

### Design

The study is designed as a cluster randomised controlled trial and process evaluation involving 32 schools to determine the effectiveness and cost effectiveness of the Healthy Lifestyles Programme in preventing childhood obesity (as assessed using BMI SDS) at 24 months.

#### Recruitment process

Schools will be recruited through presentations at the Devon and Plymouth Association of Primary Heads and at the individual local learning community meetings for head teachers grouped within a specific area in the southwest of England. Further presentations will also be made at the county conferences for deputy head teachers and primary school leads for Personal, Social and Health Education (PSHE) as necessary. These presentations will be followed up with staff meetings at individual primary schools who have shown an interest in the trial. All state primary and junior schools with children in single Year 5 groups, based in the southwest of England will be invited to participate. We will seek to ensure that at least half of the schools we recruit have 19% or more pupils eligible for free school meals, to reflect the national average. Participants will be children in Year 5 (9 to 10 year olds), and baseline assessments will be made at the beginning of the autumn term, prior to schools being randomised to control or intervention. The programme of activities takes place over the spring and summer term of Year 5 and the autumn Term of Year 6.

**Figure 1:**
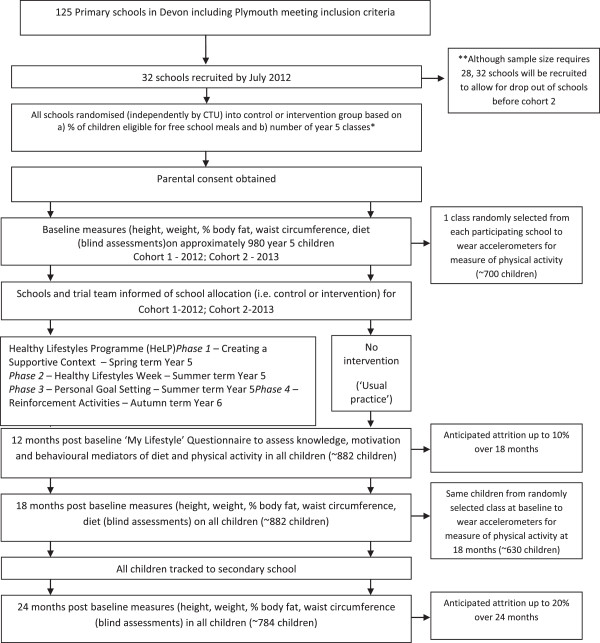
Planned flow of participants through the Healthy Lifestyles Programme randomised controlled trial.

Figure 1 shows the flow of participants through the trial.

#### Randomisation procedure

For practical reasons half of the control schools will be randomised into cohort 1 and will enter the study in year 1 (2012), and half will be randomised to cohort 2 and will enter the study in year 2 (2013). Group and cohort randomisation will be performed by a member of staff in the Peninsula Clinical Trials Unit (PenCTU) who is not involved with the trial immediately after all schools have been recruited.

The random sequence of allocation of schools to intervention or control will be computer-generated and stratified by (i) the proportion of children eligible for free school meals (<19%, ≥19%) and (ii) school size (one Year 5 class, >1 Year 5 class). Following the completion of baseline measures in each cohort the trial manager will be informed of the school’s allocated group.

### Ethical approval and consent

Ethical Approval was obtained from the Peninsula College of Medicine and Dentistry in March 2012 (reference number 12/03/140).

#### Consent

##### Families

Information sheets will be sent to parents directly from the school and will include the rationale for the study and will emphasise the importance of healthy growth. The information sheet will explain that if parents want to opt their child out of the programme, they need to return a form within two weeks, otherwise consent will be inferred. The class teacher will give verbal daily reminders to the children to ensure that they and their parents read the information sheet. This ‘opt out’ approach has been used in other cluster randomised clinical trials [33,34] and was agreed to by the Ethics Committee on the basis of the low risk of adverse effects of the intervention. Alternative arrangements for children not participating, are made in consultation with the teacher and parents, and are based on what would be best for that child.

##### Children

When anthropometric measures (height, weight, waist circumference, body fat) are taken at baseline, 18 and 24 months children have the option to decline if they so wish at the time of measurement. At baseline and 18 months measurements are carried out during a general Maths lesson on measurement. During this lesson, children are taken out of the class one at a time for a blind assessor to take the measures in private. The scales used to weigh and calculate percent body fat give a print out of the readings, thereby ensuring that children are not able to read and therefore, possibly discuss, their own results*.*

### Intervention

HeLP is a multi-component four phase programme which takes place over three school terms (the spring and summer term of Year 5 and the autumn term of Year 6) and aims to deliver a general healthy lifestyle message encouraging a healthy energy balance. Piloting has demonstrated that the children found it useful to focus on changing three specific behaviour patterns related to energy intake and expenditure: a decrease in the consumption of sweetened fizzy drinks; increasing the ratio of healthy to unhealthy snacks; and a reduction in screen-based activities. Phase 1, *Creating a Supportive Context,* aims to establish relationships and raise awareness of the programme, setting the foundation for the successful delivery of subsequent components. Phase 2 is the intensive *Healthy Lifestyles Week* involving education lessons and interactive drama activities delivered by a local drama group. Phase 3 involves the children *setting personal goals* based on the HeLP messages with their parents and phase 4 *reinforces* all the messages and knowledge and understanding using a range of activities (see Table 1).

**Table 1:** Intervention phases, change targets, behaviour change techniques and the method and agent of delivery

**Intervention phase**	**Change targets**	**Behaviour change techniques**	**Method (frequency and duration) and agent of delivery**	
**Phase 1**	Establish relationships with schools, children and families	Provide information on behaviour-health link	Whole school assembly (1)	HeLP coordinators
**Creating a supportive context**	Raise awareness and increase knowledge	Provide information on health-behaviour link	(20 minutes)	HeLP coordinators
Spring term (Year 5)	Promote positive attitudes and norms towards healthy eating and physical activity	Modelling/demonstrating behaviour	Newsletter articles (3)	Class teacher
January-March	Increase self-efficacy for behaviour change	Prompt identification as a role model	(Over the spring term)	Professional sportsmen/dancers
		Provide information on behaviour-health link	Literacy lesson (to create HeLP rap/poem) (1)	Class teachers/HeLP coordinator/Drama group
		Skill building	Activity workshops (2) (parents observe)	
			(1.5 hours)	
			Parents’ evening (1) involving child performances (1 hour)	
**Phase 2**	Strengthen relationships with schools, children and families	Provide information on health behaviour link	^a^PSHE lessons (5) (morning)	Class teacher
**Intensive Healthy Lifestyles Week -** one week	Increase knowledge	Problem solving/barrier identification Modelling/demonstrating behaviour	(1 hour)	Drama group
Summer term	Increase self-awareness	Prompt identification as a role model	^b^Drama (5)	
(Year 5)	Increase self-efficacy	Communication skills training	(afternoon)	
April-June	Develop communication and problem solving skills	Teach to use prompts and cues	(forum theatre; role play; food tasting, discussions, games, etcetera) (2 hours)	
	Increase social support (school, peer and family)			
**Phase 3**	Increase awareness of own behaviour	Self-monitoring	Self-reflection questionnaire (1) (40 minutes)	HeLP Coordinator/Class teacher
**Personal Goal Setting with Parental Support-** goals set during week following drama	Increase self-efficacy for change	Goal setting (behaviour)	Goal setting sheet to go home to parents to complete with child (1) (10 minutes)	HeLP Coordinator/Parents
Summer term	Develop planning skills	Problem solving/barrier identification	1:1 goal setting interview (1) (goals sent home to parents)	HeLP coordinator
(Year 5)	Increase parental support	Plan social support	(10 minutes)	HeLP coordinator/Drama group
June-July		Provide information on where and when to perform a behaviour	Parent’s evening (1) (child involvement - Forum Theatre) (1 hour)	
		Agree behavioural contract		
		Prompt identification as a role model		
**Phase 4**	Increase self-awareness and prioritise healthy goals.	Provide information on health behaviour link	Newsletter articles (1)	HeLP coordinator
**Reinforcement Activities**	Consolidate social support.	Prompt self-monitoring	(over the Autumn term)	Drama group
Autumn term	Develop monitoring and coping skills	Prompt intention formation	Whole school assembly (1)	Drama group
(Year 6)	Increase parental support	Follow up prompts	(20 mins)	Class teacher
September-December		Prompt practice	Drama workshop (1) (1 hour)	Children to all other year groups in the school
		Prompt review of behavioural goals	*PSHE lesson (1) (1 hour)	HeLP coordinator
		Prompt barrier identification and resolution	Class to deliver assembly about the project to rest of school (1) (20 mins)	
		Coping plans	(parents invited to attend)	
			1 to 1 goal supporting interview to discuss facilitators/barriers and to plan new coping strategies (1)	
			(10 minutes)	
			(renewed goals sent home to parents)	

^a^PSHE, Personal, Social and Health Education.

^b^The drama framework includes four characters, each represented by one of the actors, whose attributes relate to the three key behaviours. Children choose which of the characters they most resemble and then work with that actor to help the character learn to change the behaviour.

Throughout the programme the children are encouraged to find acceptable activity and dietary replacements in order to maintain a healthy energy balance. HeLP includes a range of behaviour change techniques (BCTs) designed to enhance relevant information, motivation and behavioural skills [35,36]. The Programme utilises accessible and engaging delivery methods that are compatible with the existing school curriculum as well as providing several opportunities for parental engagement. Table 1 shows each phase of HeLP, the change targets, BCTs used and the method and agent of delivery.

Our hypothesis is that targeting information, motivation and behavioural skills will lead to improvements in diet and physical activity thus preventing excessive weight gain and that these processes may be moderated by gender, weight status, socioeconomic circumstances and school size.

### Intervention fidelity

Three manuals have been developed to accompany this complex intervention. The ‘Intervention Manual’ describes and defines the programme components for each phase of delivery. The ‘HeLP Trainers’ Manual’ details the training for delivery personnel (HeLP coordinators, actors and drama facilitators). The ‘Observer’s Fidelity Manual’ incorporates a checklist that has been created to ascertain how and to what extent the intervention has been delivered as specified.

This will allow fidelity checking to occur by the trial manager, who will observe 20% of intervention components in each school. In addition to these checks, existing school practices will be carefully characterised and recorded using a predetermined checklist of possible school level mediators of childhood obesity. School activities which may affect diet and/or physical activity (PA) behaviours (for example, number of hours PA, healthy school status, provision of tuck shop, Personal, Social, Health and Education curriculum, etcetera) will be documented for both control and intervention schools.

#### Timing of assessments

Children will be assessed on four occasions, at baseline (all measures), 12 months (mediating variables only) at 18 months (all measures) and 24 months (anthropometric only) Baseline measures for Cohort 1 will begin in September 2012 and for Cohort 2 will take place in September 2013:

1. At baseline (prior to randomisation), between September and December 2012 (2013 for Cohort 2), when the children are in their first term of Year 5.

2. At 12 months post-baseline, in November 2013 (November 2014 for cohort 2).

3. At 18 months the My Lifestyle Questionnaire is administered to understand possible mediating variables, between June and July 2014 (June to July 2015 for cohort 2) when the children are finishing Year 6.

4. At 24 months post-baseline, between September and December 2014 (September to December 2015 cohort 2) when the children are in Year 7 at secondary school.

#### Methods for dealing with loss to follow-up

In the pilot trial involving 201 children, attrition rates were very low; only 8% of children were lost to follow-up at 24 months, with equal numbers retained in both control and intervention schools [25]. Our experience has shown that key to keeping children and schools involved is a combination of the fostering of trusting relationships, helping the participants to feel engaged in the research process and providing incentives. Control schools will be offered a donation of £1000 in recognition of any extra burden once the 24-month measures are completed and are offered all the intervention materials from the trial at the end of their participation (these materials include lesson plans and activities, which help schools to meet curriculum targets for Personal Social and Health Education and Science). Children in both intervention and control schools will receive a £20 voucher for completion of the 24-month measures. The value of the incentive was determined in consultation with a group of children in the piloting phases.

#### Outcome measurements

Assessments will be undertaken by HeLP Coordinators and blinded outcome assessors who will have completed enhanced criminal records bureau (CRB) checks as required for those working with children in the United Kingdom. The HeLP Coordinators will not be told which schools have been allocated to which arm of the trial until after baseline measures have been completed. Anthropometric measures at baseline, 18 and 24 months will be taken by assessors blind to group allocation.

#### Anthropometric outcomes

The primary outcome is BMI SDS at 24 months, which will be compared between the control and intervention groups. Height will be measured using a SECA stadiometer (Hamburg, Germany), recorded to an accuracy of 1 mm. Weight will be measured using the Tanita Body Composition Analyser SC-330 (U.K. Ltd., Middlesex, U.K.). Weight will be recorded to within 0.1 kg and children are asked to take off their shoes and socks. BMI is calculated and converted to centiles using the software package LMS, developed by Cole [37].

Categorisations of underweight, normal, obese or overweight will be made based on the definitions from Cole *et al*. [38].

Percent body fat will be estimated from leg-to-leg bioelectric impedance analysis (Tanita Body Composition Analyser SC-330) and converted to centiles using the LMS software [38] and compared to percentiles for British children [39].

Waist circumference will be measured using a non-elastic flexible tape measure, 4 cm above the umbilicus; converted to centiles using the LMS software and compared to the waist circumference percentiles for British children [40].

All anthropometric outcomes will be assessed at baseline, 18 and 24 months.

#### Behavioural outcomes

Physical activity will be assessed using the GENEActiv accelerometer. One randomly selected class from each school will be asked to wear a GENEActiv accelerometer (http://www.geneactiv.co.uk) [41] a watch worn around the wrist during waking and sleeping hours over seven consecutive days. GENEActiv data will be uploaded onto a PC and analysed using the GENEActiv Software (http://www.geneactiv.co.uk) [41]. Output measures will include total daily volume of physical activity and mean daily time spent in sedentary, low, moderate and vigorous intensity physical activity, with thresholds for the classification of activity intensity taken from recent research undertaken using the GENEActiv accelerometers [42,43]. How (sporadically or in bouts) and when activity is accumulated will also be determined. Where possible the GENEActivs will be charged and initialised on a Monday and given out to each child on a Tuesday and will be collected the following Wednesday. The GENEActivs will be shown to children in groups of 10 with verbal instructions given in these groups. The GENEActivs are waterproof and children will be asked to wear them all day and night.

Food intake will be assessed using the adapted version of the validated Food Intake Questionnaire (FIQ) a questionnaire specifically developed to asses change in children’s dietary habits [44]. The FIQ asks children about the food and beverages they consumed the previous day and allows an estimation of the number of health and unhealthy food and drink items to be determined. Children complete the FIQ twice in order to obtain a weekday and weekend food intake. Results are combined and weighted to calculate the mean number of healthy snacks, energy dense snacks, positive and negative foods consumed per day.

These behavioural outcomes will be assessed at baseline and at 18 months.

#### Moderating and mediating variables

Potential mediators will be assessed using a lifestyles questionnaire (‘My Lifestyle Questionnaire’) developed by the applicants as part of the process evaluation for the exploratory trial of HeLP based on the Information, Motivation and Behavoural skills Model [45] to capture possible regulatory processes that may mediate change in physical activity and diet. The items in the questionnaire assess (i) knowledge, (ii) individual motivations and cognitions, (iii) use of behaviour change techniques (BCTs), and (v) child and parental mediating behaviours that may affect levels of physical activity and diet.

The ‘My Lifestyle Questionnaire’ has been developed using the process evaluation data from the exploratory trial and two validated scales. The first is the Social Support for Diet and Exercise Behaviours Questionnaire [46], which was developed and validated in the United States to determine perceived social support for healthy diet and activity behaviours in children, and the second is a validated self-efficacy scale for diet behaviours in US primary school children [47]. These modified questions, specific to the HeLP intervention, have been piloted in the early stages of the project and found to be feasible and acceptable to children and teachers in both control and intervention schools.

Possible moderating variables (individual level SES, weight status, number of Year 5 classes and gender) will be taken from baseline data.

All questionnaires are delivered as a class activity led by the HeLP Coordinator and supported by the class teacher and Learning Support Assistants. Children sit in their literacy groups to ensure that appropriate help and guidance can be given as effectively as possible.

### Process evaluation

A process evaluation will be conducted in intervention schools to provide insight into the way HeLP worked; information on intervention uptake, delivery and experience will be collected from children, teachers and families. Delivery and uptake will be determined by assessing child and parental attendance at events and adherence to, and engagement with, HeLP. Criteria for assessing engagement for each child are: i) active participation in 90% of HeLP activities (observation); ii) parental agreement of goals (parental signature and indication of parental support); and iii) child understanding of the energy balance concept (‘My Lifestyle’ Questionnaire’). Twenty percent of activities for each intervention school will be observed, and detailed field notes taken, to determine the ‘intensity’ of the intervention components delivered; the ‘engagement’ of pupils, teachers, and parents; as well as how well the HeLP Coordinators and actors deliver the intervention. In addition, quantitative data on child and parental participation will be recorded. Qualitative interviews and focus groups will be conducted to identify barriers and facilitators to participation as well as understand the experience of participating at an individual, family and school level. Schools or children who withdraw from the intervention will be invited to participate in an exit interview/debrief with the principal investigator (PI) (KW).

Purposeful sampling will be used to identify participants for focus groups and interviews. A sampling frame has been developed for children, families and schools, sampling by level of engagement and socioeconomic status. Focus groups will be held with the children, and interviews will be conducted with parents and teachers. Approximately 14 focus groups with children (up to eight per group) and between 24 and 40 interviews with parents and teachers will be conducted.

### Statistical analyses

Throughout the analysis, emphasis will be placed on estimation rather than hypothesis testing. Where hypothesis tests are carried out, these will be at the 5% level for primary and secondary outcomes, and the 1% level for interaction terms. No adjustment for multiple analyses will be made as such adjustment methods are too conservative when outcomes are positively correlated, as they would be in this trial. However, all analyses will be planned *a priori* and reported in full.

The reporting and presentation of this trial will be in accordance with the CONSORT guidelines for cluster randomised trials [42], with the primary comparative analysis being conducted on an intention-to-treat basis. Descriptive statistics will be used to assess any marked baseline differences in demographics or outcome measures between the two groups, taking clustering into account. Comparisons of outcome measures will be undertaken at 18 and 24 months for all available measures. Comparisons of binary outcomes will be expressed as odds ratios with 95% confidence intervals and comparisons of continuous outcomes as mean differences together with 95% confidence intervals. Between-group comparisons will be made using random effects regression analysis (weighted by clusters), taking account of the hierarchical nature of the study design and allowing for adjustment by eligibility to receive free school meals, a proxy for socio-economic class, and school size, as well as important individual level baseline covariates (for example, age, sex) and baseline individual outcome values (where relevant). Sensitivity analysis, making different assumptions such as ‘best’ and ‘worst’ case scenarios, as well as imputation models of missing data, will be conducted.

Although the trial is not powered to detect the influence of mediating and moderating factors on children’s BMI, we will explore possible interactions in the following secondary analyses: (i) interaction terms will be examined to investigate possible differences in intervention effects on the primary outcome by gender, SES, baseline BMI and number of Year 5 classes; (ii) individual child estimate of engagement with HeLP will be determined and a comparison between children who meet the criteria for engagement versus those who do not will be undertaken to assess ‘per protocol’ effectiveness; (iii) a mediational analysis exploring whether the effect of the intervention on the primary and secondary outcomes is mediated by knowledge; attitudes; norms; self-efficacy; perceived environment and social support; and use of regulation techniques and behaviours relating to the physical activity and diet using the analytic framework recommended for RCTs will also be undertaken [43].

### Economic evaluation

An economic evaluation will be undertaken to estimate the incremental cost-effectiveness of HeLP compared to usual practice, from the perspective of the NHS/third party payer, with other perspectives for the public sector, and the participants, explored in sensitivity analyses. Assessment of cost-effectiveness will involve a within-trial economic analysis and a model-based economic evaluation to assess the longer term cost-effectiveness of HeLP.

Within-trial analyses will provide a robust estimate of the resource use and costs associated with delivery of the HeLP intervention, based on regular reporting of resource use (for example, trial report forms) by those hosting and delivering the intervention. Resource use data (in physical units, for example, staff time, consumables) will be combined with appropriate unit costs, to estimate a mean incremental cost per school, and a mean incremental cost per child. Cost-effectiveness analysis (CEA) will be presented against effectiveness outcomes for the study (for example, cost per unit change in BMI, cost per change in proportion of overweight/obese). Results from the trial-based CEA will be presented in a disaggregated way in a tabular format that is useful to decision-makers. Uncertainty in estimates will be explored using detailed sensitivity analyses.

The assessment of cost-effectiveness over a longer term time horizon will be via a model-based evaluation to explore the broader policy context of the effects of the intervention and to present a policy-relevant CEA (for example, cost per life-year, cost per quality-adjusted life-year). The modelling framework will link effectiveness outcomes to weight status over time (child to adult), and the impact of weight status to future health outcomes (for example, prevention of adult overweight/obesity, diabetes, coronary heart disease), with costs and quality-adjusted life-years (QALYs) for health outcomes over time informing the CEA. Modelling methods will be transparent, will be informed by systematic review to populate the model, and will follow guidelines for good practice in modelling for health technology assessment [48].

### Qualitative data analyses

All interviews and focus groups will be audio-taped and transcribed verbatim. Transcribed data will be managed using NVivo software which will also support the coding and analytical processes. All of the transcripts will be read and re-read in order to gain an overall understanding of participants' views and experiences. As this process evaluation is partially driven by predetermined concepts, a framework analysis approach [49] will be adopted for the analysis and interpretation of emergent themes.

### Power and sample size

To have 90% power, and two-sided 5% significance level, to detect a between-group difference in BMI SDS of 0.25 units at 24 months, assuming a standard deviation of 1.3 and adjusting for baseline BMI SDS (assuming within-person correlation of 0.8 [50]) and allowing for an attrition rate of 20%, requires a total of 952 children to be recruited. Data from approximately 35,000 National Child Measurement Programme (NCMP) records for Year 6 children in Devon has been used to estimate the likely school intraclass correlation coefficient (ICC) (95% CI: 0.005 to 0.017). We anticipate an effect size of at least 0.19 standard deviation units (that is, difference of 0.25 units in BMI SDS) in our primary outcome; a difference of 0.25 units in BMI SDS has been shown to be a meaningful change impacting on improvement in adiposity and metabolic health [51]. Furthermore the mean between-group difference (intervention minus control) in BMI SDS of approximately −0.2 units (95% CI −0.5 to 0.1) at 24 months in our exploratory trial demonstrates such an effect size to be plausible. Statistical efficiency will be maximised by analysing BMI SDS while adjusting for baseline values. Although the correlation between baseline and 24 months BMI SDS was 0.93 (95% CI: 0.92 to 0.96) in the exploratory trial, more conservative estimates have been used in our sample size calculations. Similarly, while we had an 8% attrition rate in our exploratory trial, we have allowed for an attrition rate of 20%.

Table 2 illustrates the range of likely effect sizes detectable, based on recruiting 980 children, across plausible values for the ICC within person correlation coefficient and attrition rates. Under these various scenarios, our sample size would allow us to detect an effect size ranging at best from 0.14 to 0.25 standard deviation units at worst (that is, between-group difference in mean BMI SDS of 0.18 to 0.32).

**Table 2:** Sample size scenarios based on differing assumptions

	**ICC**	**Within person correlation coefficient**	**Attrition rate**	**Effect size detectable**	
				**Number of SD units**	**Difference in BMI SDS**^**a**^
**Base case**	**0.02**	**0.8**	**20%**	**0.19**	**0.25**
*Vary ICC*	0	0.8	20%	0.14	0.18
	0.02	0.8	20%	0.19	0.25
	0.05	0.8	20%	0.25	0.32
*Vary correlation between baseline and 24 months BMI SDS*	0.02	0.75	20%	0.21	0.27
	0.02	0.8	20%	0.19	0.25
	0.02	0.85	20%	0.17	0.22
	0.02	0.9	20%	0.14	0.18
*Vary attrition rate*	0.02	0.8	10%	0.18	0.23
	0.02	0.8	20%	0.19	0.25
	0.02	0.8	30%	0.20	0.26

^a^Between-group difference in BMI SDS at 24-month follow-up.

All figures based on 90% power; two-sided, 5% significance level; mean cluster size of 35; intraperson correlation of 0.8. BMI SDS, body mass index standard deviation scores; ICC, intraclass correlation coefficient.

## Discussion

This paper describes the protocol for a cluster RCT to determine the effectiveness and cost effectiveness of a school-based intervention (HeLP) aimed at preventing obesity in children.

The prevalence of childhood obesity has increased three-fold (from 5% to 17%) in the last 30 years and is linked with increasing prevalence of type 2 diabetes, hypertension and atherosclerosis (http://www.cdc.gov/growthcharts) [52]. Moreover the current high prevalence of adult obesity suggests that all young people regardless of weight status are at risk of adult obesity [53]. In England, one third of 10 to 11 year olds are overweight or obese and the distribution of body mass index has shifted in a skewed fashion such that the heaviest children have become heavier [8,9]. Childhood obesity has significant adverse physical and psychological effects in childhood and tracks strongly into adult life. Children who are overweight in the primary school years are reported to be almost 20 times more likely to be overweight as teenagers compared to young children of normal weight [54]. Behavioural treatments of established obesity in both children and adults are generally of limited effectiveness [55] and it is now recognised that early prevention to avoid unhealthy behaviours are critical for all children and adolescents and not just those already overweight [57].

HeLP has been developed and piloted according to the MRC guidance for developing and evaluating complex interventions [32]. HeLP is based on the Information Motivation Behavioural (IMB) Skills Model which proposes that adequate information, motivation and behavioural skills are essential to achieve behavioural change. It has been systematically developed in a UK population, working with teachers, parents and children using an Intervention Mapping approach [55]. This approach has guided the linking of theory to specific behaviour change targets and their associated behaviour change techniques and delivery methods [35,57]. Programme development was founded on a recognition that children’s behaviours are shaped not only by their individual decisions but by their peer group at school, by the school environment and, importantly, by their families. Consequently, the intervention is designed to instigate change at different levels. As well as prompting individual children to advocate and plan behavioural change, the intervention aims to change the whole school environment using creative delivery methods specifically designed to engage all children and teachers within the school as well using multiple approaches to engage families.

The systematic theoretical underpinning of ‘HeLP’ will allow additional questions to be addressed regarding the effects of moderators and operation of mediators on outcomes and understand the extent to which some or all of the psychological and behavioural variables (mediators) explain the outcomes in weight status. The process evaluation will provide additional insight into why the intervention was successful or not and allow us to assess ease of delivery and the experience of the participants (teachers, children and parents). An understanding of these issues will enable any necessary post-trial modifications or remodelling in order to enhance the effectiveness of HeLP prior to its larger scale roll-out.

The primary outcome measure for HeLP is BMI SDS at 24 months post baseline (assessed in the first term of Year 7 when the children have moved on to secondary school). The timing of the main outcome measure has been chosen to allow us to understand whether any effect on outcome is sustained into secondary school when the children are no longer in the same class. Our analyses of Devon National School Measurement data, in line with the national data [9], show that the observed increase in childhood obesity reflects a shift in the population distribution, not a change in the shape of the distribution and HeLP takes a whole population approach to obesity prevention [36].

The results from this trial will provide evidence regarding the effectiveness and cost effectiveness of a novel intervention which seeks to create supportive environments within the school and at home to prevent obesity in school children. Any amendments or updates to this protocol will be lodged with the journal such that it links them to this protocol document. This will allow all future trial publications and conclusions to be assessed against the extent to which we have adhered to the protocol.

## Trial status

Since receiving funding and ethical approval for the study we have recruited the trial manager, administrator and two HeLP coordinators and a physical activity coordinator. Presentations have been made at the Devon and Plymouth Associations of Primary Heads as well as a number of county learning community meetings for head teachers. Fifty schools have expressed an interest in participating in the trial of which 41 meet the inclusion criteria.

## Abbreviations

BCT: Behaviour change techniques; BMI: Body mass index; CEA: Cost effectiveness analysis; CI: Confidence interval; ICC: Intraclass correlation coefficient; PI: Principal investigator; NICE: National Institute for Clinical Effectiveness; NCMP: National Child Measurement Programme; PenCTU: Peninsula Clinical Trials Unit; QALY: Quality-adjusted life-year; RCT: Randomised controlled trial; SD: Standard deviation; SDS: Standard deviation scores; PA: Physical activity; PSHE: Personal social and health education; WC: Waist circumference.

## Competing interests

The authors declare that they have no competing interests.

## Authors’ contributions

KW, JL, RTo, RTa, CG, SC, and SL completed the pilot phases and exploratory trial of HeLP. KW and JL wrote the first draft of this paper. SC and RTA completed the sample size calculations and with KW, JL, SL and MH developed the quantitative analysis protocol. JL, SD and CA developed the qualitative methods and analysis protocol for data collected from these. CG developed the economic evaluation methods and associated analysis protocol. VP, WD and ED provided advice on recruitment strategies, participant information literature and school and parental engagement activities. All authors contributed to the final version of the paper and will be responsible for conducting the trial. All authors read and approved the final manuscript.
